# Management of the pterygoid plate in orthognathic surgery: a narrative review

**DOI:** 10.1186/s40902-025-00487-4

**Published:** 2025-10-10

**Authors:** Ji Young Hwang, Dae Seok Hwang, Borim Choi

**Affiliations:** 1 Department of Oral and Maxillofacial Surgery, School of Dentistry, Pusan National University, Yangsan, Republic of Korea; 2 Dental Research Institute, Pusan National University Dental Hospital, Yangsan, 50612 Republic of Korea

**Keywords:** Le Fort osteotomy, Orthognathic surgery, Maxilla surgery, Pterygoid plate, Complications

## Abstract

**Background:**

The most critical factor for successfully performing setback and superior/posterior impaction of the maxilla in orthognathic surgery is securing sufficient space. This space can be achieved through pterygoid plate fracture, removal, or grinding. However, most studies to date have provided limited investigation into the management of the pterygoid process itself.

**Main text:**

Safe manipulation of the pterygoid plate demands a precise understanding of the surrounding anatomy. The course of the internal maxillary artery demonstrates substantial anatomical and ethnic variability. In the relationship with the lateral pterygoid muscle, lateral positioning of the artery is more common in Asian populations, and symmetric courses occur more frequently than asymmetric ones. Preservation of the descending palatine artery helps maintain perfusion of the maxilla, although ligation may be indicated in certain situations. The risk of vascular injury increases when the osteotome is placed more than 15 mm superior to the most inferior point of the pterygomaxillary junction. To avoid high-level fractures, the osteotome should be positioned with its lower end at the base of the pterygomaxillary fissure and its tip directed superiorly. These anatomical considerations are essential for selecting the appropriate technique among available options, including fracture, removal, and grinding.

**Conclusions:**

All three methods of pterygoid plate management—fracture, removal, and grinding—are all effective methods that provide skeletal stability and demonstrate no significant differences in complications including bleeding. Optimal technique selection should be guided by detailed anatomical knowledge, patient-specific factors, and intraoperative findings to maximize surgical safety and effectiveness.

## Background

Dentoskeletal deformities are often treated with a combination of orthodontic therapy and surgical repositioning of the facial bones. This approach improves both function—such as occlusion and jaw movement—and facial esthetics by enhancing facial balance. The Le Fort I osteotomy is one of the most commonly performed procedures in this context. Recently, the Le Fort I osteotomy has become the standard treatment approach for a variety of conditions, including mandibular prognathism, maxillary retrusion, facial asymmetry, and gummy smile.

Among these, maxillary setback and superior/posterior impaction through Le Fort I osteotomy has recently become established as a primary surgical approach in cases requiring maxillary setback, replacing earlier techniques such as maxillary anterior segmental osteotomy [1–3]. During maxillary setback and superior/posterior impaction, surgeons frequently encounter bony interference between the posterior maxilla and the pterygoid plate [1, 4–6]. The removal of such bony interference is essential to achieve sufficient maxillary movement which enables both satisfactory esthetic outcomes and stable occlusion. To address this interference, maxillary tuberosity removal, pterygoid plate fracture and removal have been introduced as effective surgical techniques.

Management of the pterygoid process is essential during Le Fort I osteotomy, as it plays a critical role in enabling sufficient posterior repositioning of the maxilla and ensuring postoperative stability. Nevertheless, most existing studies have primarily focused on the separation of the pterygomaxillary junction (PMJ) [7–14], with limited attention given to the surgical management of the pterygoid process itself and its associated complications.

Therefore, this narrative review aims to explore the surgical approaches to the pterygoid process, grounded in its anatomical considerations, and to evaluate potential complications such as intraoperative bleeding, unintended fractures, and fixation instability. Relevant literature was identified through a non-systematic search of electronic databases, including PubMed and Google Scholar, using keywords such as “pterygoid,” “orthognathic,” “Le Fort,” “maxillary,” and “setback.” The anatomical considerations, surgical techniques, and complications were extracted and analyzed. The initial search indicated 729 studies, of which 44 studies were included for analysis.

## Main text

### Anatomical consideration

#### Anatomical relationship between the internal maxillary artery and the lateral pterygoid muscle

The internal maxillary artery (IMA) is recognized as one of the major sources of hemorrhagic complications during orthognathic surgery [15–17]. In particular, laceration of the artery caused by sharp bony fragments from a detached pterygoid plate can result in life-threatening outcomes depending on the site of injury [15, 18, 19]. Therefore, precise anatomical knowledge of the maxillary artery is essential for safe surgical manipulation in the posterior maxillary region.

Anatomically, the maxillary artery is traditionally divided into three segments: (1) the mandibular portion, (2) the pterygoid (or muscular) portion, and (3) the pterygopalatine portion; the mandibular portion lies posterior to the lower border of the lateral pterygoid muscle; the pterygoid portion may pass either deep to or superficial to the lateral pterygoid muscle; the pterygopalatine portion courses through the pterygopalatine fossa.

While bleeding from terminal branches such as the descending palatine artery can usually be managed effectively through electrocauterization or vessel ligation, injury to the more deeply located pterygoid segment may necessitate ligation of the external carotid artery—a more invasive and potentially hazardous intervention. Consequently, understanding the anatomical course of the IMA, particularly during pterygoid plate manipulation, is of paramount importance. Especially, the second segment, the pterygoid portion, remains one of the most anatomically controversial segments of the artery. Typically, the artery courses along the surface of the lateral pterygoid muscle and passes between its two heads before entering the pterygopalatine fossa.

Although the IMA is generally protected by the overlying lateral pterygoid muscle during surgical procedures, the positional relationship between the artery and the muscle has been shown to vary among different populations.

A cadaveric study conducted in Korea involving 100 Asian specimens reported that in 82% of cases, the maxillary artery traveled lateral to the lateral pterygoid muscle; 11% exhibited an intramuscular course, and 7% passed medially [20]. Consistent findings were reported in another CT-based study of Korean individuals, where the IMA was positioned lateral to the lateral pterygoid muscle in 82% of cases [21]. Similarly, a study targeting Japanese cadavers found that 90.4% of IMAs coursed laterally to the muscle, and approximately 89% showed bilateral symmetry [22]. Rischmüller and Meiring examined African and European populations, reporting that 53.93% of IMAs in African individuals passed along the lateral surface of the muscle, compared to 67–69% in Europeans. Moreover, bilateral symmetry was observed in only about 50% of African individuals [23]. Following cadaveric investigations, radiographic studies were subsequently conducted. Gulses et al. conducted a radiographic assessment of the IMA trajectory in a Turkish cohort, noting that 68.42% of arteries followed a superficial course relative to the muscle, with bilateral symmetry in 78.9% of subjects [24]. Overall, lateral positioning of the IMA appeared to be more frequent in Asian populations than in other ethnic groups, and symmetric courses were generally more common than asymmetric ones [25].

Taken together, these findings underscore the significant anatomical and ethnic variability in the course of the IMA, which must be taken into consideration during posterior maxillary surgical interventions.

#### Anatomical relationship between the internal maxillary artery and the pterygomaxillary junction

Brisk hemorrhage during Le Fort I osteotomy most commonly occurs during mobilization of the PMJ, where displacement may result in injury to the IMA or its terminal branches. Accordingly, numerous studies have investigated the anatomical relationship between the PMJ and the IMA. Most studies have focused on evaluating the distance between the IMA and the most inferior point of the PMJ. Based on these anatomical measurements, recommendations have been proposed to guide the safe and optimal positioning of the osteotome during Le Fort I osteotomy, with the aim of minimizing the risk of vascular injury.

Manchella et al. conducted a computed tomography (CT)–based study in an Australian population and reported that the IMA was located, on average, 29 ± 3 mm superior to the most inferior point of the PMJ [26]. Bendrihem and Vacher, analyzing CT scans of a French population, found that the IMA was located 18.22 ± 3.79 mm superior to the PMJ at the average height and 18.85 ± 3.26 mm at its maximal height [27]. Apinhasmit et al., in a cadaveric study of Thai individuals, reported that the IMA entered the pterygopalatine fossa (PPF) approximately 23.5 ± 2.5 mm above the lowest point of the PMJ [28]. However, this study had the limitation that the measurements were not obtained in a strictly vertical plane. In a cadaveric study of South Korean individuals, Choi and Park measured the vertical distance from the lowest point of the PMJ to the point where the posterior superior alveolar artery entered its foramen, which was found to be 15.2 ± 2.4 mm [29]. Based on their findings, they recommended that the osteotomy edge should not extend beyond 20 mm superior to the PMJ to minimize the risk of IMA injury.

Although some variability was noted across studies depending on measurement criteria, methodological approaches, and ethnic populations, a common conclusion can be drawn: the positioning and direction of the osteotome during PMJ separation are critical. In general, placement of the osteotome more than 18 mm superior to the inferior border of the PMJ may significantly increase the risk of IMA injury during PMJ displacement. Consequently, it may be inferred that placing the osteotome more than 15 mm superior to the PMJ may increase the risk of injury to the internal maxillary artery during PMJ displacement.

#### Preservation or ligation of the descending palatine artery

Approximately 80% of intraoperative bleeding during Le Fort I osteotomy is attributed to injury of the descending palatine artery (DPA), making it a major source of hemorrhage [15, 30]. The DPA has also been recognized as a significant contributor to hemorrhage during procedures involving fracture or removal of the pterygoid process. Therefore, a thorough understanding of the anatomical location, course, and management strategies of the DPA is essential to minimize the risk of intraoperative hemorrhage during management of the pterygoid process.

The management of the DPA during Le Fort I osteotomy remains controversial, with ongoing debate regarding whether to preserve or ligate the vessel. Surgeons who advocate for DPA preservation argue that maintaining the integrity of the artery helps minimize the risk of mucosal and bony ischemia or ischemic necrosis. In contrast, those who favor DPA ligation base their rationale on the assumption of sufficient collateral circulation, citing benefits such as improved maxillary mobilization, easier manipulation of the pterygoid region, and more effective control of postoperative bleeding. In practice, DPA ligation is commonly achieved using a ligation clip or cauterization, and hemorrhage is controlled with a hemostatic matrix combined with thrombin.

Numerous studies have investigated the vascular supply and potential complications associated with ligation of the DPA. Early investigations, including animal models, reported conflicting conclusions regarding the extent of perfusion compromise following DPA ligation. However, the resulting ischemia and reduction in blood flow have generally been regarded as transient and limited, with minimal influence on bone healing [31–35]. In clinical studies, Dodson et al. conducted a randomized controlled trial involving 34 patients, comparing outcomes between DPA ligation and preservation groups [36]. Intraoperative gingival blood flow was assessed using laser Doppler flowmetry, and the results indicated that DPA ligation was not associated with significant changes in anterior maxillary gingival blood flow during Le Fort I osteotomy. Gauthier et al. further demonstrated the presence of substitute vascularization by injecting colored latex into cadaveric specimens and evaluating the collateral circulation [37]. Their findings revealed that, following DPA ligation, branches of the ascending palatine artery and ascending pharyngeal artery provided compensatory blood supply to the soft palate. While the soft palate exhibited adequate perfusion, the mucosa overlying the bony palate appeared relatively paler, suggesting a decrease in vascular density in that region.

Taken together, ligation of the DPA is not generally associated with severe adverse outcomes. However, preserving the DPA may help maintain maxillary perfusion and reduce the risk of postoperative hemorrhage-related complications. Preservation of the DPA is recommended whenever feasible, and routine ligation of the DPA during pterygoid plate management should be approached with caution.

### Management of the pterygoid plate

#### Intentional fracture of the pterygoid plate

The pterygoid plate arises from the base of the sphenoid bone and constitutes the posterior boundary of the pterygopalatine fossa. Due to the bony interferences between the maxilla and the pterygoid plate, resection of the maxillary tuberosity or the pterygoid plate is necessary for maxillary setback.

Historically, due to concerns about hemorrhage during the management of the pterygoid plate, grinding of the maxillary tuberosity was preferred [1, 38, 39]. Since the mid-2000s, studies have emerged aiming to make maxillary setback and superior/posterior impaction more predictable, and currently, the management of the pterygoid plate has become the most preferred approach.

Intentional pterygoid plate fracture has advantages over other methods such as anterior edge resection or maxillary tuberosity removal, including reduced operative time and easier application. During pterygoid plate fracture, it is essential to confirm the mobility of the fractured segment. Incomplete fractures can interfere with posterior-superior movement of the maxilla.

Pterygoid plate fractures are classified by fracture level: low-level fractures occur at the osteotomy cut level; high-level fractures occur superior to the cut level. One of the most important precautions is to prevent HLF. HLF inadvertently developing at the root of the pterygoid plates may also propagate across the skull base or to the orbit and, in turn, may account for peripheral neuromorbidity [8, 9, 11, 17, 40]. Improper osteotome position and angulation have been cited as major causes in the literature. The osteotome should be placed as inferiorly as possible, angled medially and anteriorly, and must not be directed superiorly.

During PMJ separation, unintentional changes to the pterygoid plate may occur. Robinson and Henry classified these fracture patterns into four types [11]: (1) intact pterygoid plates with complete separation of the plates and tuberosity; (2) fracture at the osteotomy level (LLF); (3) fracture higher than the osteotomy (HLF); (4) multiple fractures at both levels (MLF). Such unintended fractures occur in 30–75% of cases [8, 10, 11, 41]. As with intentional pterygoid plate fracture, proper osteotome positioning is crucial for preventing HLF [9, 11, 29]. The osteotome should be positioned with its lower end at the base of the pterygomaxillary fissure and the tip directed superiorly. To minimize posterosuperior pressure on the pterygoid process, the handle should be angled anteromedially. Nevertheless, serious complications due to pterygoid plate fractures are undoubtedly low, and some studies report no HLF even when fractures occur [9, 17, 26, 41, 42].

Possible complications that may arise from pterygoid plate fracture include cavernous sinus bleeding, dislocation of the fractured bone segments due to the pulling force of the lateral pterygoid muscle, and failure to achieve complete maxillary setback due to residual bony interference from incompletely fractured segments [6, 37, 41], as well as other complications previously described in the anatomical section.

Despite these risks, most orthognathic surgeries involving pterygoid plate fracture proceed without major complications such as hemorrhage, nerve injury, or airway obstruction due to edema. Furthermore, relapse is rare [5–7, 41, 43, 44]. While few studies have focused on bone healing, most confirm appropriate healing at the pterygomaxillary junction [5, 6, 43].

#### Removal of the pterygoid plate

Whether to fracture or remove the pterygoid plate during maxillary setback is determined by several factors. In cases where the pterygoid plate is robust or hyperplastic and removal is technically difficult, the fracture method or maxillary tuberosity removal may be preferable to direct removal [17, 44]. If interferences between fracture segments hinder mobilization, segmental removal should be considered. When significant superior maxillary repositioning is required, the fracture method may be safer to avoid injury to the internal maxillary artery. A comparative study between pterygoid plate fracture and removal reported no significant difference in complication rates and postoperative stability [44]. The authors recommended upper pterygoid plate removal over fracture when more than 5 mm of superior or more than 3 mm of maxillary setback movement is planned [44]. However, in the case of maxillary setback, the loss of bone contact may increase the risk of nonunion [45]. Therefore, appropriate interventions, such as bone grafting, should be considered when necessary.

Successful setback and superior/posterior impaction of the maxilla depends not solely on the choice between pterygoid plate fracture and removal. Rather, it requires a comprehensive understanding of the pterygoid process anatomy, surrounding vasculature, and the extent of movement required.

#### Maxillary tuberosity and pterygoid plate grinding

In the past, during maxillary setback procedures, maxillary tuberosity grinding was often preferred over pterygoid plate fracture due to concerns about hemorrhage. This technique preserved the pterygoid plate and the greater palatine neurovascular bundle, thereby reducing complications related to arterial injury—both intraoperative and postoperative bleeding—and minimizing disturbances to the Eustachian tube [1, 5, 38, 39, 46].

However, more recent comparative studies have demonstrated no significant differences between the management of the pterygoid plate and the removal of the maxillary tuberosity in terms of postoperative relapse, skeletal stability, or bone healing [4, 5, 47]. On the contrary, maxillary tuberosity removal has been associated with significantly longer operative times and increased hemorrhage. Furthermore, maxillary tuberosity and pterygoid plate grinding alone was insufficient to fully achieve the planned surgical objectives. With advancements in hemostatic agents and surgical techniques, the management of the pterygoid plate has now become the most preferred approach in contemporary practice.

### Complications

There are relatively few studies that have directly compared pterygoid plate management with maxillary tuberosity removal. Xiang et al. compared intentional pterygoid plate fracture and maxillary tuberosity removal in Le Fort I osteotomy [4]. While no significant postoperative relapse was observed in either group, both intraoperative blood loss and operative time were significantly greater in the maxillary tuberosity removal group. In a similar study conducted by Han and Hwang, maxillary tuberosity removal was initially performed in all patients, and pterygoid plate fracture was subsequently carried out only when bony interference persisted [5]. In this study, postoperative stability was assessed based on a threshold of 2 mm of maxillary setback, with no significant difference in relapse observed between the groups. Regarding bone healing, no significant differences were found between the plate management and maxillary tuberosity removal groups.

In a study by Lee et al., skeletal stability was compared between the pterygoid plate fracture and removal groups, and no significant differences in postoperative skeletal changes were identified [44]. Moon et al. investigated postoperative complications between patients who underwent pterygoid plate fracture versus removal [45]. No significant difference in hemorrhage was observed, although one case of transient hearing disturbance was reported in the removal group. The symptom resolved spontaneously within 1 month, likely due to resolution of middle ear edema and recovery of associated muscle function. Similarly, other studies have reported mild and transient auditory changes within the first postoperative week following bimaxillary surgery, which were generally self-limiting and clinically negligible.

Additional studies addressing either pterygoid plate management or maxillary tuberosity removal independently have consistently reported no significant postoperative relapse in terms of skeletal stability.

## Conclusions

In orthognathic surgery, precise management of the pterygoid plate is essential for accurate maxillary positioning. Surgical techniques such as grinding, fracture, and removal of the pterygoid plate are all effective methods that provide skeletal stability and demonstrate no significant differences in complications including bleeding.

## Data Availability

No datasets were generated or analysed during the current study.
